# Protein Kinases and Transcription Factors Activation in Response to UV-Radiation of Skin: Implications for Carcinogenesis

**DOI:** 10.3390/ijms13010142

**Published:** 2011-12-23

**Authors:** César López-Camarillo, Elena Aréchaga Ocampo, Mavil López Casamichana, Carlos Pérez-Plasencia, Elizbeth Álvarez-Sánchez, Laurence A. Marchat

**Affiliations:** 1 Genomics Sciences Program, Oncogenomics and Cancer Proteomics Laboratory, University Autonomous of Mexico City, Av. San Lorenzo 290, 03100, Mexico; E-Mails: mavil.lopez@uacm.edu.mx (M.L.-C.); maria.alvarez@uacm.edu.mx (E.Á.-S.); 2 Carcinogenesis Laboratory, National Institute of Cancerology, Av. Saint Fernando 22, 14080, México; E-Mail: earechagao@incan.edu.mx; 3 Massive Sequencing Unit, National Institute of Cancerology, Av. Saint Fernando 22, 14080, México; E-Mail: carlos.pplas@campus.iztacala.unam.mx; 4 Genomics Laboratory, FES-I, UBIMED, National Autonomous University of Mexico, Av. De los Barrios 1, 54090, México; 5 Biotechnology Program, Institutional Program of Molecular Biomedicine, National School of Medicine and Homeopathy of the National Polytechnic Institute, Guillermo Massieu Helguera 239, 07320, Mexico; E-Mail: lmarchat@ipn.mx

**Keywords:** ultraviolet radiation, skin cancer, skin photoaging, p38, MAPK, JNK, ERK1/2, ATM, SKR kinases, NF-κB, AP-1, NRF2 transcription factors

## Abstract

Solar ultraviolet (UV) radiation is an important environmental factor that leads to immune suppression, inflammation, photoaging, and skin carcinogenesis. Here, we reviewed the specific signal transduction pathways and transcription factors involved in the cellular response to UV-irradiation. Increasing experimental data supporting a role for p38, MAPK, JNK, ERK1/2, and ATM kinases in the response network to UV exposure is discussed. We also reviewed the participation of NF-κB, AP-1, and NRF2 transcription factors in the control of gene expression after UV-irradiation. In addition, we discussed the promising chemotherapeutic intervention of transcription factors signaling by natural compounds. Finally, we focused on the review of data emerging from the use of DNA microarray technology to determine changes in global gene expression in keratinocytes and melanocytes in response to UV treatment. Efforts to obtain a comprehensive portrait of the transcriptional events regulating photodamage of intact human epidermis after UV exposure reveals the existence of novel factors participating in UV-induced cell death. Progress in understanding the multitude of mechanisms induced by UV-irradiation could lead to the potential use of protein kinases and novel proteins as specific targets for the prevention and control of skin cancer.

## 1. Introduction

Ultraviolet radiation from sunlight is an environmental factor that has a variety of physiological and biological effects, including immune suppression, cellular aging, DNA damage and initiation of apoptosis [1,2]. In addition, UV radiation promotes the generation of reactive oxygen species (ROS) that can cause oxidative damage, and ultimately lead to tumor formation. Solar UV radiation is also a very prominent environmental toxic agent and it is known to be one of the main causes of human skin cancers, such as cutaneous, malignant melanomas and non-melanoma tumors that include basal cell carcinomas and squamous cell carcinomas [3,4]. UV is classified in three bands according to wavelength: UV-A (320–400 nm), UV-B (290–320 nm) and UV-C (<290 nm). UV-A is also subclassified into UV-A1 (340–400 nm) and UV-A2 (320–340 nm). The UV component of the sunlight reaching the earth surface (daylight UV) consists in 90% UV-A and 10% UV-B, while UV-C and almost all UV-B (80–90%) are absorbed by ozone layer [5,6]. The biological effect induced by UV depends largely on wavelength [7,8]. The hypothesis is that UV wavelength-specific “action spectrum” is stemmed from distinct direct damages to various biomolecules. Conjugated bonds, linear repeats or ring structures in organic molecules absorb shortwave UV radiation (200–300 nm). Proteins containing tryptophan or tyrosine can therefore absorb UV radiation and start up photochemical reactions causing alterations in signaling pathways. UV-A and UV-B portion has a strong carcinogenic effect on the skin [9–11] to directly or indirectly induce DNA damage leading to genomic mutations and modified gene expression [8,12,13]. UV-C has also been described as inductor of genetic and cellular modifications in experimentally assays [14].

Exposure to UV-A leads to oxidative DNA modifications including the formation of 8-hydroxyguanine, DNA-protein cross-linking, abasic sites, and DNA strand breaks [7,15]. In contrast, the shorter wavelengths, UV-B and UV-C, directly damage DNA, causing the formation of cyclobutane pyrimidine dimers or 6–4 photoproducts [11,14]. In addition to DNA damage, UV enhances the production of reactive oxygen species (ROS) and induces activation of specific signal transduction pathways, resulting in altered gene expression that induce senescence, cell cycle arrest and cell death [16–18].

## 2. Signal Transduction Pathways in the Cellular Response to UV Radiation

**Figure 1:**
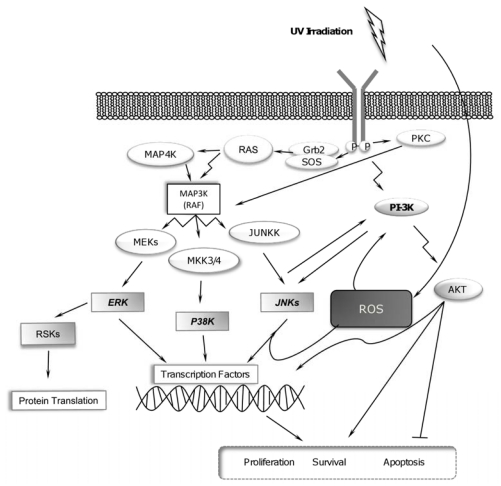
Regulation of proliferation and survival pathways by protein kinases activation in response to UV radiation. Mitogen-Activated Protein Kinases (MAPK) family and Phosphoinositide 3-Kinases (PI-3K) signaling activation as an outcome of Epidermal Growth Factor Receptor (EGFR) activation and/or increase in intracellular Reactive Oxygen Species (ROS) levels resulting from UV radiation. MAPK and PI-3K pathways can regulate each other in response to UV radiation; however extracellular signal-regulated kinase (ERK), p38K and c-Jun NH_2_-Terminal Kinases (JNK) activate specific transcription factors, which regulate the expression of genes participating in proliferation or cell survival. PI-3K activates AKT, which leads to induction or repression of cell survival and apoptosis. UV radiation is also able to activate protein kinases to regulate protein translation through ERK pathway activation.

UV radiation is one of the most important kinds of environmental stresses for skin damage. Exposure to UV is known to induce clustering of some kinds of cell-surface receptors and to transducer some cell survival and proliferation signals [19,20]. Skin cells respond to damage by triggering the activation of cell surface receptors, such as epidermal growth factor receptor (EGFR) and tumor necrosis factor receptor (TNFR) [19,21], and activate intracellular signaling mediated by SRC family of tyrosine kinases [22], the small guanosine triphosphate binding proteins, RAS and RAC [23], phosphatidylinositol 3-kinase (PI-3K) [24], mitogen-activated protein kinases (MAPKs) pathways [25], ataxia telangectasia mutated (ATM) [26] and ribosomal S6 kinases (RSK) [27]. Activation of intracellular signaling pathways in response to UV irradiation induces various transcription factors that transactivate genes involved in DNA repair, DNA synthesis, transcription and cell cycle regulation [17] (Figure 1). The effects to UV radiation on gene expression could be classified as early or late events, according to the activation of specific genes and proteins; these UV responses are important in determining cell fate, such as growth arrest, apoptosis and survival. UV radiation activates different signal pathways in a time, dose and wavelength-specific manner [17,28].

### 2.1. UV-Radiation Activates MAPK Signaling Pathways

MAPKs belong to the serine/threonine protein kinases family that is activated by several stimuli, including UV irradiation [25]. The MAPK signaling pathways are, in general, subdivided into three different pathways, namely the extracellular signal-regulated kinases (ERK), p38 MAPK (p38 kinase), and c-Jun NH_2_-terminal kinases (JNK) signaling pathways [29], which are activated by dual phosphorylation of threonine and tyrosine, at TX-Y motifs within their activation loops. Each member of this family can be activated in response to different stimuli and they also have specific intracellular targets. The ERK cascade is generally activated by mitogenic stimuli, and evidence suggested that it mediates both cell proliferation and survival, whereas the JNK and p38 kinase pathways are activated in response to cellular stress, and appear to exert both protective and pro-apoptotic effects [30–32]. UV radiation could activate the three subgroups of the MAPK family. In fact, some reports suggest that MAPK pathway is the central event that links UV-induced intracellular signal to the nuclear response and modify DNA damage-originated cellular response [25]. Previous reports suggested that induction of distinct MAPK signaling pathways by UV is wavelength-specific. Apparently, UV-A induces stronger activation of ERK but JNK is principally activated by UV-C, while p38 kinases could be activated in response to any UV wavelength to modify DNA damage response [25,33]. Because the UV-C component of sunlight is filtered by the atmosphere and does not reach the earth’s surface, here we will mainly focus in the studies about activation of MAPK pathway in response to UV-A and UV-B radiation as they are more relevant for skin carcinogenesis.

Activation of MAPK by UV may result from the EGFR-tyrosine kinase (TK) domain activation, although the precise mechanism of UV irradiation induced-EGFR activation has not been elucidated yet. It has been suggested that UV irradiation might lead clustering of receptors in cell membrane surface in order to allow autophosphorylation and activation of intracellular signaling pathways [20,34]. Other works indicated that EGFR activation by UV irradiation might depend on the ROS generation, particularly the increased intracellular hydrogen peroxide (H_2_O_2_), which has been associated with phosphorylation of the EGFR-TK domain [20]. The activation of three MAP kinases was prevented by antioxidant reagents, suggesting that an oxidative signal initiates these responses [35]. Recently, Xu and co-workers reported that UV irradiation induce EGFR nuclear translocation depending of phosphorylation of tyrosines in TK domain in human keratinocytes [36]. The biological function of nuclear EGFR remains unclear. It has been suggested that EGFR nuclear may phosphorylate nuclear proteins and transactivate genes [37,38]. These observations suggested that nuclear EGFR in UV radiated cells might regulate cell proliferation. ERK pathway is primarily activated in response to activation of growth factors, suggesting that activation of this pathway might result from EGFR activation in response to UV-A, whereas activation of JNK and p38 kinases is directly induced by stress, including UV-B and UV-C radiation [39]. However, when p38 kinase activation by UV-B exposure is mediated by EGFR, it is associated with protection against apoptosis [40], while ERK and JNK activation in response to UV-B radiation could be regulated by protein kinase C (PKC) and is associated to cell proliferation [41]. Phosphorylation of JNK and p38 kinase by UV-B irradiation might result in down regulation of ERK signaling [41].

In human keratinocytes, UV-A and UV-B stimulate the activation MAPK pathways [42], particularly, ERK1, ERK2 and JNK kinases, but p38 is fewer stimulated by UV-A. Interestingly, MAPK and transcription factors as AP-1 are not activated in mouse keratinocytes in response to UV-A and UV-B, which suggested that a human component, distinct from kinases, could regulate the MAPK activation in human keratinocytes. UV-A radiation stimulates ERK signaling pathway in response to activation of EGFR, SRC, RAS and RAF proteins in NCTC 2544 keratinocytes [43]. UV-B mainly acts through JNK and has a weak effect on ERK in human epidermal keratinocytes [44]. In normal human epidermal melanocytes, UV-A increases ERK phosphorylation but it has no effect on the activation of JNK and p38 kinases [45]. MAPK pathways activation is a mechanism for causing early-response gene expression by controlling the activities of several transcription factors. Activated MAPKs translocate to the nucleus, where they phosphorylate target transcription factors, including AP-1, NF-κB, and p53. Phosphorylation of transcription factors and regulatory cell survival proteins is mediated by activation of effector kinases downstream of the MAPK signaling cascade. UV-B stimulates the activation of JNK1, p90 ribosomal S6 kinase 2 (RSK2) and mitogen-and stress-activated protein kinase (MSK1), which are downstream of ERK and p38 kinase, respectively [29].

### 2.2. UV Radiation-Induced Phosphatases

MAPKs are inactivated by dephosphorylation induced by mitogen-activated protein kinase phosphatases (MKPs), thus regulating the physiological outcome of signaling in response to UV radiation. MAPKs are the central element of the signaling cascade that links UV-induced intracellular signals to the nuclear responses by phosphorylated transcription factors [25]. MAPK are known to be differentially activated depending on dose and wavelength of UV radiation [25,33] and phosphatases activation in response to UV stimulus may be an important mechanism of regulation of cell response to UV irradiation. Phosphatase 2A (PP2A) is a major protein serine/threonine phosphatase that participates in many signaling pathways in mammalian cells [46]. UV-induced PP2A activation resulted in down-regulation of p38 MAPK and Akt activities, and diminished apoptosis in human keratinocytes. These findings suggest that PP2A may maintain the equilibrium between cell survival and cell death in UV irradiation-keratinocytes response in p38 MAPK/Akt-dependent manner [47]. The c-JUN-JNK and p53-p38 kinase pathways may regulate activation of wild-type p53-induced phosphatase (Wip1) in response to UV-radiation. Wip1 dephosphorylates several proteins, included MAPK activated by stress stimuli. Apparently, p38 kinase-activated p53 is more efficient to activate Wip1, than c-Jun-JNK. As the pathways execute somewhat different cellular responses with regard to cell cycle arrest and apoptosis in response to UV irradiation, Wip1 may regulate negatively UV irradiation-induced apoptosis by dephosphorylate some kinases [48]. EGFR activation in UV radiated-cells may be result of intercross of EGFR in cell membrane or due to phosphorylation induced by ROS. Protein-tyrosine phosphatase (PTP) family can dephosphorylate EGFR in order to negatively regulate its activity. PTP contain an active site cysteine residue, which is required for phosphohydrolase activity. This active site cysteine is highly susceptible to oxidation by ROS, particularly by H_2_O_2_ [49]. This mechanism could be important for activation of EGFR by UV irradiation. Therefore, inactivation of PTP activity as a consequence of intracellular ROS production may regulate activation of EGFR by UV radiation [50,51]. Activation of kinases and phosphatases in UV irradiated cells may represent a feedback mechanism to regulate the ultimate fate of the cell.

### 2.3. UV Radiation Induces PI-3K Pathway for Promoting Cell Survival

One of the best characterized effectors of cell survival and proliferation is the phosphoinositide 3-kinases (PI-3K) pathway [52]. When active, PI-3K converts phosphatidylinositol-(4,5)-bisphosphate (PIP2) into phosphatidylinositol-(3,4,5)-trisphosphate (PIP3). PIP3, in turn, binds the pleckstrin homology (PH) domain of Akt, stimulating its kinase activity. Akt, also known as protein kinase B, is catalytically activated by phosphorylation at Thr308 and Ser473 [53]. Recently, many new downstream targets of Akt have been identified and shown to promote proliferation by facilitating cell cycle progression, or raise survival by transcription-dependent or transcription-independent means [54] or by phosphorylation of other proteins that affect apoptosis and cell proliferation [53]. UV-B exposure may stimulate PI-3K activity more strongly that exposure to UV-A [55]. UV-B irradiation induces survival signals via activation of PI-3K-Akt pathway [56]; this activation could be a direct effect of UV or via activation of EGFR [51]. This activity was associated with overexpression of c-FOS and phosphorylation of Akt and p70^s6k^. UV induces suppression of cell death through phosphorylation of apoptotic proteins and Akt activates transcription factors in order to stimulate genes that promote survival [53]. In other words, the activation of receptor cell membrane surface, *i.e.*, clustering of receptors (dimerization) in response to UV-B radiation, results in PI-3K activation.

The activation of PI-3K-Akt pathway is directly activated by EGFR activation in response to UV-B irradiation, but activation of this pathway is weaker in response to UV-A radiation. Activation of PI-3K-Akt pathway can induce phosphorylation and activation of other kinases, such as p70^s6k^ and p90^RSK^, to promote synthesis and activation of transcription factors for the expression of genes associated with cell survival [55].

### 2.4. RSK Activation and UV Radiation

UV-A-induced signal transduction leads to the activation of a complex network of downstream effector molecules, including RSK p70^s6k^ and p90^RSK^, also known as MAPKAP-K1, which in turn phosphorylate the 40S ribosomal protein S6, resulting in an increase of the translational machinery component [57]. p90^RSK^ is a member of the family of 90-kDa ribosomal S6 kinases, which are phosphorylated by ERKs. p90^RSK^ is involved in signal transduction leading to proliferation, differentiation, and apoptosis [58]. In response to UV-A irradiation, EGFR activates p70^s6k^ and p90^RSK^ kinases through the activation of ERK, but not JNK and p38 [33,59]. The activation of RSKs can occur by the MAPK pathways or PI-3K according to the phosphorylation site of each kinase, *i.e.*, MAPK and Akt phosphorylate different RSK protein residues [58]. p70^s6k^ activation in response to UV-A irradiation is mediated by the activation of EGFR and PI-3K-Akt. The effects on phosphorylation and activation of Akt and p70^s6k^ may be mediated by MSK1 in response to activation of p38 and ERK, but not JNK [59]. EGFR activation in response to UV-A irradiation activates p70^s6k^ and p90^RSK^ kinases through the activation of ERK. An increased activation of basal and EGF-inducible MAPK, p90^RSK^ and p70^s6k^ is associated with the activation of AP-1 in cell transformation and tumor promotion [60].

## 3. ATM and UV Radiation

ATM is a member of the PI-3K family [61]; it is a serine-threonine protein kinase with a carboxy-terminal domain similar to the catalytic subunit of PI-3K. In particular, ATM binds to and phosphorylates the tumor suppressor p53 in response to DNA double-strand breaks (DSB) [62]. Both p53 and ATM function as tumor suppressors [63]. Experimental knockout of both *p53* and *atm* genes resulted in a marked acceleration of tumorigenesis in comparison with that observed with a knockout of only one of these genes [64]. ATM does not only regulate the response to DSB (see section 4) but it also plays an important role in the response to oxidative stress and for modulating cell growth through growth factor receptors [26,65]. ATM is activated by UV-A and is involved in the cellular decision to trigger p53- and JNK-dependent apoptosis after radiation exposure [26]. ATM is also activated in response to ionizing radiation [66]. Unlike UV-A, UV-C does not activate ATM [26]. Instead, UV-C appears to stimulate ATM-related (ATR), a kinase structurally and functionally related to ATM, that may function as a sensor of DNA damage to activate JNK and p53 signaling leading to apoptosis [67]. Thus, these two related kinases, ATM and ATR, may be selective sensors of different types of cellular stress, with ATM responsive to UV-A and ionizing radiation and ATR responsive to UV-C irradiation.

Activation of survival pathways and cell proliferation induced by diverse UV wavelengths occurs through a complex network of protein kinases (Table 1). The activation of MAPK pathway by UV-radiation, not only has association with activation of membrane receptors, such as EGFR, but it is also associated with increased intracellular ROS levels. UV radiation can promote cell survival through PI-3K-Akt pathway, in a dependent or independent way of EGFR activation, according to UV wavelength, time exposure and dose. MAPK pathway could regulate the PI-3K activity in response to UV-irradiation independently of EGFR activation. Some investigators have reported that the activation of AP-1 and NF-κB transcription factors by UV radiation is mediated through membrane-associated signaling proteins and is not dependent on a nuclear signal [68]. DNA damage is directly induced by UV exposure, and is an important mechanism for UV-induced tumor initiation [69]. Cell response to DNA damage includes cell cycle G1-arrest through ATM and ATR activation, using or not the p53 signal transduction pathways. Therefore the signaling from nucleus in response to UV-irradiation could be regulated by p53, which is found to be a negative regulator of AP-1 and NF-κB activation in response to UV radiation. This mechanism is linked, on one hand, with the inhibition of JNK and p38 pathway but not ERK, and on the other hand, with the overexpression of PTEN, which negatively regulates activation of PI-3K-Akt pathway. Both events are related to the low activation of AP-1 and NF-κB [8].

## 4. Role of Transcription Factors in Response to UV Radiation

Exposure to UV leads to premature aging of the skin and it also increases the risk of acquiring cancer. UV-B radiation of cells induces the activation of specific transcription factors, which in turn regulate the expression of a number of genes termed the “UV response genes”. Two transcription factors that are activated in this way are the activator protein-1 (AP-1) and NF-κB [9]. The study of these two transcription factors and the cross-talk between them in response to UV-B exposure may help to the development of new chemopreventive strategies for the prevention of UV-B induced skin carcinogenesis.

### 4.1. The Nuclear Factor NF-κB

NF-κB represents a family of transcription factors involved in the regulation of inflammation, immune response, development, cellular survival, and cell proliferation. They are the effectors of a signaling system that is responsive to a large number of stimuli, including inflammatory cytokines recognized by TNFR, and pathogens and virus recognized by Toll-like receptors (TLRs), and receptors of the NACHT-LRR (leucine-rich repeat) families [70]. NF-κB also regulates the expression of genes outside of the immune system. In the skin, NF-κB plays a role in epidermal homeostasis. The range of stimuli activating NF-κB is extensive and continuously growing, which emphasizes its key role in transcriptional responses. Notably, NF-κB has important functions in the transcriptional response to cytotoxic agents like chemotherapeutic drugs, oxidative and physical stress, UV and ionizing radiations. In addition, recent work has highlighted its role in development and physiology of tissues including mammary gland, bone, and skin. Thus, the NF-κB system operates on transient or short timescales, relevant to inflammation and immune responses, and on longer-term timescales during cell differentiation and organ formation [71].

NF-κB exists in the cytoplasm as homodimers or heterodimers of a family of structurally related DNA-binding proteins [72]. In mammals, NF-κB family members are represented by five “Rel” proteins: RelA (p65), c-Bel, RelB, and two “NF-κB” proteins NF-κB1 (p50/p105) and NF-κB2 (p52/p100), which dimerize in almost any combination to activate transcription. NF-κB 1 and NF-κB2 are synthesized as large precursors (p105 and p100) and processed to generate the mature transcriptional active proteins. Only RelA, c-Rel, and RelB, but not p50 or p52, share the C-terminal transactivation domains, whereas Rel and NF-κB B proteins contain the N-terminal Rel homology domain (RHD), which is involved in dimerization, DNA binding and nuclear localization. In most cells, NF-κB is present as a latent and inactive cytoplasmic complex bound to inhibitory IκB proteins. In the canonical NF-κB activation pathway, the activity of NF-κB is negatively regulated by interaction with inhibitory IκB factors (IκBα, IκBβ, IκBγ, IκBδ, IκBɛ, and Bcl-3), which prevents DNA binding and promotes cytoplasmic accumulation of the interacting p50/RelA dimeric complex. IκB proteins have different affinities for diverse NF-κB dimers, which greatly contributes to the complex fine-tuning of NF-κB functions [73]. Positive regulation of NF-κB is controlled by the IκB kinase (IKK) complex, which is in turn activated by cytokines, growth factors, antigen receptors, MAPKs and other kinases. IKK complex, which contains the IKKα and IKKβ catalytic subunits and the regulatory scaffold sensing protein called NF-κB essential modulator (NEMO), phosphorylates IκB proteins leading to their ubiquitination and subsequent proteasome-dependent degradation [72]. The resulting nuclear accumulation of the released NF-κB complexes leads to transcriptional activation of targets genes. Inputs for the classical pathway include TNFR1/2, TCR and BCR, TLR/IL-1R, among others. In the alternative pathway, activation of IKKα occurs by the activity of NF-κB inducing kinase (NIK) that leads to the phosphorylation and processing of p100 bound to RelB, generating p52/RelB heterodimers, which in turn translocate to nucleus to activate gene transcription. Activating signals for the alternative pathway include ligation of LTβR, BAFFR, and CD40R [74]. An important issue about the cellular response to UV irradiation is the existence of crosstalk between NF-κB and specific signaling pathways, including the MAPKs, such as JNKs, ERKs and p38 MAP kinases. These observations have been extensively reviewed [75], therefore they will not be discussed here.

UV-B radiation of mammalian cells induces the activation of several transcription factors including NF-κB and the subsequent transcription of genes [9]. Here, we reviewed relevant aspects about NF-κB activation in the context of UV-induced stress. The original evidence for NF-κB regulation by UV comes from the initial observation that UV exposure of culture cells induces transcription from the long terminal repeat (LTR) promoter of human immunodeficiency virus 1 (HIV-1), the collagenase gene and the cellular oncogene *fos*. Transcriptional activation of HIV-1 depends on the two NF-κB-binding sites located in the LTR (-105 and -79 nucleotide positions), whereas the heterodimer of JUN and FOS (AP-1) appears to bind to the collagenase enhancer, and the serum response factors p67 and p62 bind to *c-fos* gene promoter. In addition, DNA-binding activities of the factors recognizing the HIV-1 and collagenase enhancers were augmented in extracts from UV-treated cells. These data indicated that UV, like TNFα or IL-1α, targets NF-κB to the nucleus. However, the canonical NF-κB activation mechanism described above does not occur in UV-induced early phase (12 h). NF-κB-dependent HIV-LTR activation required lower doses of UV-C (2 J/m^2^) in cells from patients with Xeroderma pigmentosum group A (XPA) than in cells from healthy human individuals (20 J/m^2^), which supports the idea that DNA damage is required for proper NF-κB activation [76].

**Figure 2:**
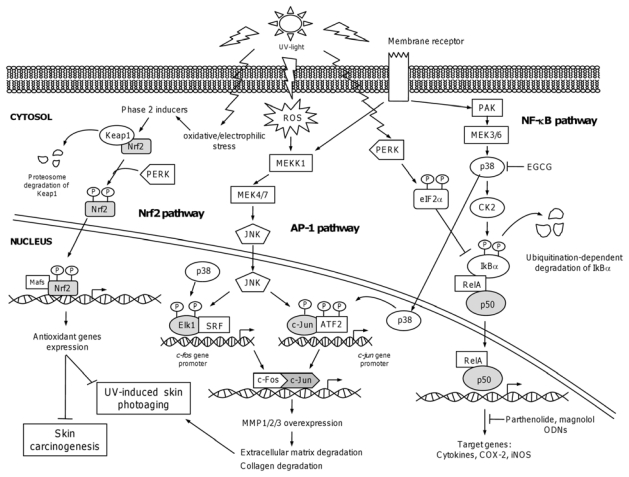
Signaling pathways leading to transcription factors activation in response to UV-radiation. UV-induced Nuclear Factor kappa B (NF-κB) pathway activation depends on phosphorylation of IκBα by Casein Kinase II (CK2) through the activation of p38-MAP kinase. Phosphorylation-induced degradation of IκBα releases NF-κB subunits RelA/p50, which enter into nucleus to activate gene transcription of targets genes. UV-induced phosphorylation of Eukaryotic Translation Initiation Factor 2α (eIF2α) inhibits *de novo* IκBα translation, whereas the existing IκBα is degraded, leading to NF-κB activation. The chemopreventive inhibition of NF-κB by natural compounds parthenolide and magnolol, as well as p38 blocking by (−)-Epigallocatechin 3-gallate (EGCG) is depicted. In AP-1 pathway, UV-induced activation of membrane receptors leads to the activation of MEKK, c-Jun NH_2_-Terminal Kinases (JNK), and p38. MAPKs phosphorylate transcription factors leading to increased expression of AP-1. In Nrf2 pathway, Nrf2 acts as a sensor for oxidative and electrophilic stress. In these conditions, phase 2 inducers cause the modification of Keap1 and/or phosphorylation of Nrf2 resulting in the release of Nrf2 from Keap1. Nrf2 is stabilized and translocate to the nucleus, where it heterodimerizes with MAF proteins, to bind to the antioxidant responsive element which leads to activation of cytoprotective genes.

Other studies showed that UV-C radiation results in degradation of NF-κB inhibitor IκBα, nuclear translocation of RelA (p65), and induction of NF-κB DNA-binding activity. It was suggested that UV-C induces phosphorylation-independent degradation of IκBs by the ubiquitin and proteasome pathway. Interestingly, expression of a catalytically inactive IKKβ mutant did not prevent NF-κB activation by UV-C, indicating that activation occurs through a mechanism independent of IKK activation [77]. UV-induced NF-κB activation depends on phosphorylation of IκBα by casein kinase II (CK2) through a mechanism that depends on the activation of p38 MAP kinase (Figure 2). Notably, inhibition of this pathway prevents UV-induced IκBα degradation and increases UV-induced cell death [78].

NF-κB activation may occur sequentially in time by different mechanisms. For instance, it has been observed that UV radiation induces a delayed and prolonged (3–20 h) activation of NF-κB, whereas TNFα exposure induced an immediate and acute (10–90 min) activation. It was reported that UV causes IκB degradation in an early phase (30 min to 6 h), which does not involve the phosphorylation of IκB by IκB kinase (IKK complex); while a late mechanism established in cultured cells beyond 15 h requires the phosphorylation of IκBα, and the DNA damage-induced release of IL-1α which in turn binds to its receptor and activates the IKK complex. This late phase of NF-κB activation appears to be due to DNA damage-dependent activation of the IKK complex. In contrast, early phase activation was independent of DNA damage [79]. UV-B radiation induction of NF-κB activity independently from chromosomal DNA damage has been previously observed using UV irradiated cell-free cytosolic extracts [80]. In agreement with these observations, the UV response does not require a signal generated in the nucleus and is not dependent on DNA damage. Enucleated cells are fully responsive to UV irradiation through NF-κB induction and activation of additional key signaling events [68].

A novel mechanism mediating the early phase-NF-κB activation by UV radiation involved an endoplasmic reticulum-stress-induced translational inhibition pathway [81]. UV-induced phosphorylation of the eukaryotic translation initiation factor 2α (eIF2α), inhibits *de novo* IκB translation, whereas the existing IκB is degraded through the ubiquitin-dependent proteasomal pathway, leading to NF-κB activation in mouse embryo fibroblasts. In this model, inhibition of IκB mRNA translation was initiated by UV-C-induced (0.03 kJ/m^2^) phosphorylation of eIF2α through activation of the protein kinase like endoplasmic reticulum kinase (PERK) leading to early phase activation of NF-κB. Other studies evidenced the activation of NF-κB via a non-canonical pathway. It was reported that both TNFα and UV-B stimuli enhanced NF-κB DNA-binding activity in normal human keratinocytes, however these events occur through different mechanisms. NF-κB response following TNFα treatment occurs by the canonical pathway and leads to IκBα phosphorylation and degradation; whereas activation of NF-κB by UV-B is independent of IκBα degradation. Moreover, TNFα or UV-B treatment results in the activation or repression of a subset of specific genes [82]. These observations provide further experimental evidence about the stimuli and cell-type dependence of NF-κB activation.

### 4.2. Chemopreventive Intervention of NF-κB Pathway

The UV induced-skin photoaging is characterized by keratinocytes proliferation and degradation of collagen fibers, leading to skin wrinkling and laxity. It is well known that NF-κB activity greatly contributes to the skin photoaging process. Therefore, it is plausible that inhibition of NF-κB pathway could directly prevent skin photoaging effects. The natural compound parthenolide, a sesquiterpene lactone from the plant feverfew (*Tanacetum parthenium*) can inhibit the gene expression mediated by NF-κB and the production of basic fibroblast growth factor (bFGF) and matrix metalloproteinase-1 (MMP-1). Notably, this compound also inhibited the UV-B-induced proliferation of keratinocytes and melanocytes in mouse skin [83,84]. Parthenolide induced apoptosis and inhibited cell proliferation and the expression of VEGF *in vitro*. Moreover, inhibition of NF-κB activity by expressing a mutant IκBα suppressed lung cancer metastasis *in vivo* [85]. Magnolol is a bioactive compound found in the Japanese whitebark Magnolia (*Magnolia ovovata*) that specifically inhibited the NF-κB-dependent transcription. In addition, external swabbing with Magnolia extracts prevented skin photoaging process in mice. Magnolol was also effective in inhibiting the production of bFGF and MMP-1 from cells overexpressing p65, a major subunit of NF-κB. Interestingly, magnolol did not affect the phosphorylation and degradation of IκBα, but it inhibited the nuclear translocation of the activated NF-κB [86]. Treatment of normal human epidermal keratinocytes with grape seed pro-anthocyanidins (GSP) inhibited UV-B-induced hydrogen peroxide, lipid peroxidation, protein oxidation, and DNA damage. GSP also inhibited UV-B-induced phosphorylation of ERK1/2, JNK, and p38. Moreover, GSP inhibited the UV-B induced activation of NF-κB/p65 through inhibition of degradation and activation of IκBα and IKKα, respectively [87]. (−)-Epigallocatechin 3-gallate (EGCG) from green tea leaves (*Camellia sinensis*) is a potent antioxidant that may have therapeutic applications in the treatment of many disorders (e.g., inflammation, cancer). It has been described that EGCG is able to inhibit p38, therefore blocking NF-κB activation and *c-fos* transcription (Figure 2). In conclusion, these findings suggest that NF-κB inhibitors and modulators are useful in preventing the skin photoaging, UV-radiation-induced oxidative stress, as well as proliferation and metastasis of tumoral cells.

Alternative strategies to target NF-κB functions have been developed for potential therapeutically intervention. Experimentally induced sunburn reactions in mice could be specifically prevented by blocking UV-induced, NF-κB-dependent gene transactivation with oligodeoxynucleotides (ODNs) containing the NF-κB *cis* element (NF-κB decoy ODNs). Local UV-induced inflammatory changes represented by cellular swelling, leukocyte infiltration, epidermal hyperplasia, and accumulation of proinflammatory cytokines IL-1, IL-6, TNFα, and VEGF were inhibited by topically applied decoy ODNs [88]. Similarly, NF-κB decoy ODNs lead to increased UV-B induced apoptosis of mouse transfected keratinocyte Pam 212 cells. In addition, cell sunburn formation was significantly enhanced by topical NF-κB decoy ODNs treatments, while UV-induced erythema was not affected [89]. These data promise for the potential application of decoy ODNs targeting NF-κB function as a new therapeutic modality for the treatment of skin diseases.

### 4.3. The Activator Protein-1: AP-1

Exposure to UV-B leads to the activation of cell surface growth factor and cytokine receptors, which induces multiple signal transduction pathways that converge to stimulate DNA binding of specific transcription factors, such as the activator protein-1 (AP-1), and subsequent modulation of gene expression [9]. The AP-1 transcription factor family is composed of homodimers or heterodimers of basic region-leucine zipper (bZIP) proteins that belong to the JUN (c-JUN, JUNB and JUND), FOS (c-FOS, FOSB, FRA-1 and FRA-2), Jun dimerization partners (JDP1 and JDP2), the activating transcription factors (ATF2, LRF1/ATF3 and B-ATF) and musculoaponeurotic fibrosarcoma (MAF) subfamilies [90]. The bZIP domains allow binding to other proteins that contain a similar bZIP domain. DNA-binding specificity and affinity is governed by the broad combinatorial possibilities resulting from the large number of AP-1 proteins, which greatly increases the spectrum of regulated genes.

AP-1 transcription factor is mainly composed of JUN, FOS and ATF dimers, which after activation by phosphorylation, mediate gene expression regulation in response to a variety of physiological and pathological stimuli, including cytokines, growth factors, hormones, stress signals, bacterial and viral infections, as well as oncogenic stimuli. In turn, AP-1 regulates a wide range of cellular processes, including cell proliferation, differentiation, survival, apoptosis and transformation [91]. These effects are mediated through activation and binding of AP-1 dimers to DNA consensus sequences, leading to expression regulation of target genes, such as collagenase-1, c-JUN, cyclin D1, p21, p19 and p16 [92]. JUN-FOS heterodimers bind to the heptamer consensus sequence known as the TPA responsive element (5′-TGA(C/G)TCA-3′), whereas Jun-ATF dimers bind with higher affinity to the consensus sequence known as the cyclic AMP responsive element (5′-TGACGTCA-3′).

Transactivation of AP-1 is required for tumor promoter-induced transformation in mouse epidermal JB6 cells and for progression in mouse and human keratinocytes. The relevant roles of AP-1 in skin cancer promotion were early described in clonal genetic variants of mouse epidermal JB6 cells that are susceptible or resistant to promotion of transformation by phorbol ester 12-*O*-tetradecanoylphorbol-13-acetate (TPA). Using constructs containing AP-1 *cis*-enhancer sequences upstream of a reporter gene, Bernstein and Colburn [93] evidenced an association between induced AP-1 function and oncogenic transformation in JB6 cells. Constitutive AP-1 DNA binding and subsequent transactivating abilities occur in malignant but not in benign mouse epidermal cell line 308, suggesting that AP-1 activation resulted in deregulation of gene expression leading to cell neoplastic transformation. In contrast, stable expression of a c-JUN deletion mutant (TAM67) in two malignant mouse epidermal cell lines blocked tumor formation in nude mice [94]. Exposure of dominant negative TAM67 under the control of the human keratin-14 promoter blocked tumor promoter-induced AP-1 transactivation and neoplastic transformation [95]. Expression of TAM67 inhibited AP-1 and NF-κB transactivation and suppresses anchorage independent growth of HPV immortalized human keratinocytes, revealing the existence of a “crosstalk” between AP-1 and NF-κB transcription factors [96]. The same group reported additional evidences suggesting that “cross-coupling” of AP-1 and NF-κB activation might contribute to the process of tumor promoter-induced transformation induced by TPA and TNFα [97]. Using okadaic acid, a prototypical non-phorbol ester skin tumor-promoting agent, in transgenic mice expressing TAM67, Thompson and coworkers [98] observed that TAM67 was able to block okadaic acid-induced skin tumor promotion, which indicates that functional AP-1 is required for TPA induced tumor promotion in multistage mouse skin carcinogenesis *in vivo*. Potential target genes responsible for TAM67 inhibition of 7,12-dimethylbenz(*a*)anthracene-TPA-induced tumorigenesis were identified using microarray expression profiling of epidermal tissues. Cyclooxygenase-2 (Cox-2/Ptgs2) and osteopontin (Opn/Spp1), which are known to be important contributors in driving carcinogenesis, were identified as TAM67-modulated genes [99]. Cooper *et al.* [100] expressed the TAM67 mutant transgene in the epidermis of SKH-1 hairless mice and bred with mice expressing an AP-1 luciferase reporter gene. Authors showed that expression of TAM67 efficiently inhibits UV-B-induced squamous cell carcinoma number and size in the SKH-1 hairless mouse, which correlated with a diminution in cyclin D1 expression. Taken altogether, these data suggested that specific signaling pathways that are inhibited by a dominant negative c-JUN are involved in both chemical and UV-B-induced skin carcinogenesis. They also evidenced that AP-1 is a good candidate target for the development of chemoprevention strategies to prevent sunlight-induced skin cancers.

Crosstalk between AP-1 subunits and MAPKs may exist. Involvement of signaling molecules in AP-1 activation has been reviewed [101]. In addition, crosstalk between AP-1 and NF-κB has been recently reviewed [9], thus they will not be discussed here. The roles of p38 and ERK on UV-B induced *c-fos* transcription factor gene expression were previously studied in the human keratinocytes cell line FL30 [102]. Using specific inhibitors, authors showed that suppression of both p38 and ERK completely blocked UV-B induced *c-fos* expression. In addition, inhibition of p38 and ERK in a squamous cell carcinoma cell line SCL-1 has been shown to inhibit UV-A and UV-B-induced matrix metalloproteinases MMP-1 (stromelysin-2) and MMP-10 (interstitial collagenase) expression both *in vitro* [103] and *in vivo* [104]. These findings identify the JNKs and p38 as important players in UV-B-induced AP-1 activation in UV-B-induced skin carcinogenesis, which argues for the identification of novel specific inhibitors as chemopreventive agents.

### 4.4. The NF-E2-Related Factor 2: Nrf2

The accumulation of ROS contributes to a wide variety of degenerative diseases including skin cancer. Cytoprotection is provided by the expression of antioxidant proteins and phase 2 detoxifying enzymes that are induced upon exposure to low levels of electrophiles or oxidative stress. These cellular antioxidants prevent the occurrence and reduce the severity and appearance of UV-induced photoaging and skin cancer. The protective mechanisms described involves the NF-E2-related factor 2 (Nrf2), which binds to a *cis-*acting sequence called the antioxidant responsive element (ARE) or electrophile responsive element within the regulatory upstream region of target cytoprotective genes coding for antioxidant proteins. Under basal conditions, Kelch-like ECH associating protein 1 (Keap1) regulates the intracellular localization of Nrf2 by direct binding to its *N*-terminal Neh2 domain [105]. Keap1 interaction with Neh2 allows the sequestration of Nrf2 in the cytosol and the enhancement of Nrf2 degradation by proteasomes, conferring the fine tuning to regulation of response [106]. During periods of oxidative/electrophilic stress, phase 2 inducers cause the modification of Keap1 (Cys151) and/or PKC phosphorylation of Keap1 (Ser40) resulting in the release of Nrf2 from Keap1. Nrf2 is stabilized and translocates to the nucleus, where it forms a complex with MAF proteins and binds to ARE motifs to promote transcription of “anti-oxidant” genes. Therefore, Nrf2 and its cytoplasmic anchor protein Keap1, regulate the cellular antioxidant response through the expression and coordinated induction of a battery of defensive genes encoding detoxifying enzymes and antioxidant proteins.

Nrf2 was first isolated as a protein closely related to p45 NF-E2. Four members of the p45 NF-E2-related proteins, p45 NF-E2, Nrf1, Nrf2, and Nrf3, have been isolated in mammals and referred to as Cap‘n’collar (CNC)-type basic leucine zipper (bZIP) transcription factors [107]. The roles of Nrf2 and phase 2 genes in chemoprevention of carcinogenesis have been well described in several tissues and organs; however only few studies have focused in the elucidation of their functions in skin [108]. Many harmful effects of UV radiation are associated with the generation of ROS, thus cellular antioxidants should act to prevent UV-induced skin damage. Keratinocyte growth factor (KGF), a potent mitogen for epithelial cells, promotes survival under stress conditions, including ROS generation. Braun and colleges [109] reported a role for KGF-regulated Nrf2 in the control of gene expression and inflammation during cutaneous wound repair *in vivo*. UV-A, but not UV-B, could activate Nrf2 in dermal fibroblasts to ensure protection of dermis against photo-oxidative stress. In addition, using dermal fibroblasts derived from *nrf2* or *keap1* gene knockout mice, they demonstrated that disruption of *nrf2* increased the number of apoptotic cells after UV-A radiation, whereas disruption of *keap1* decreased the apoptotic cell number by half as compared with wild-type animals. These observations evidenced that Nrf2-Keap1 complex has an important function in the protection of skin against UV-A irradiation. However, the role of Nrf2 in response to UV-B exposure has not been clearly demonstrated and contradictory data have been reported [110]. Only low UV-B doses (7.5 J/m^2^) could stimulate Nrf2 transcriptional activity in normal human fibroblasts. Intriguingly, high UV-B dose (20 J/m^2^) led to the nuclear exclusion of Nrf2 and down-regulation of chemoprotective gene expression [111]. In contrast, Durchdewald *et al.* [112] reported that electrophilic chemicals, but not UV radiation, could activate Nrf2 in mouse keratinocytes.

These countered points have been reconciled by observations indicating that p53 may compete with Nrf2 and suppress the Nrf2 transcription of phase 2 genes [113]. Authors proposed that if the oxidative stress causes DNA damage, a second response based on the activation of p53 takes place, which induces cell cycle arrest and/or apoptosis. p53 activation counteracts the Nrf2-induced transcription of ARE-containing promoters in antioxidant genes. Thus, it seems that a p53-dependant negative control on the Nrf2 transactivation should be activated to block the generation of a strong antioxidant intracellular environment that could dam the induction of apoptosis. Additional evidences about the role of Nrf2 in the UV response of the skin were obtained using a transgenic mice expressing an Nrf2 mutant (K5cre-caNrf2), which lacks the *N*-terminal Neh2 domain responsible for binding to the inhibitory Keap1, resulting in a specifically activation of Nrf2 in the epidermis. Results showed that Nrf2 activates the production and release of glutathione and cysteine by suprabasal keratinocytes, resulting in protection of basal cells from UV-B-induced apoptosis through activation of cytoprotective genes. In addition, Nrf2 protects keratinocytes from UV-B cytotoxicity through enhanced ROS detoxification [114].

The involvement of Nrf2 in skin cancer prevention was demonstrated by auf dem Keller *et al.* [115]. Intriguingly, authors did not find gross abnormalities in the epidermis of transgenic mice, which express a dominant-negative Nrf2 mutant in the epidermis; however, the incidence and multiplicity of chemically induced skin papillomas were enhanced. In addition, they evidenced that increased tumorigenesis results from a reduced basal expression of cytoprotective Nrf target genes, leading to accumulation of oxidative damage driving to genomic instability and neoplastic transformation. In contrast, Kawachi *et al.* [116] reported no significant differences in skin carcinogenesis between nrf2-null and wild-type mice exposed to chronic UV-B radiation. Notably, they observed that epidermal necrosis, inflammatory cell infiltration, sunburn cell formation, accumulation of oxidative DNA products and cell apoptosis after acute UV-B treatment (single dose), were more prominent in nrf2-null mice, suggesting that the Nrf2-Keap1 pathway plays an important role in protection of the skin against acute UV-B reactions.

A concerted interplay between Keap1-Nrf2-Maf signaling and other signaling pathways such as PKC, MAPKs, PI3K, PERK, p38, ERK2 and JNK1 have been reported and reviewed [117–120] and therefore will not be discussed here.

### 4.5. Chemopreventive Intervention of Nfr2-Keap1 Pathway

The chemopreventive effects of natural compounds in photo-dermatological reactions caused by oxidative stress have been well documented. Exposure to chemopreventive agents produces ROS or electrophiles and causes oxidative/electrophilic stress in cells, which induces phase 2 detoxification enzymes and antioxidant enzymes [121]. Treatment of mouse and human keratinocytes with sulforaphane, a natural compound found in broccoli, Brussels sprouts or cabbages, increases glutathione and phase 2 enzymes protecting against oxidative stress generated by UV radiation. Moreover, topical application of sulforaphane-containing broccoli sprout extracts protects against UV-B (30 mJ/cm^2^)-induced skin carcinogenesis in SKH-1 high-risk mice, suggesting that this approach constitutes a promising strategy for protecting skin against tumor formation after exposure to UV radiation [122]. In other studies, quercetin, a polyphenol found in fruits, vegetables, leaves and grains, protected human keratinocyte HaCaT cells from UV-A induced apoptosis and DNA damage by enhanced accumulation of Nrf2, which leads to elevating intracellular antioxidative activity and reduced production of ROS [123].

### 4.6. Factor of Activated T Cells (NFAT) is Activated in Response to UV Radiation

NFAT is a family of nuclear transcription factors that are regulated by calcium/calcineurin signals [124]. They were identified for the first time as transcription factors of genes associated to immune response and play an important role during T cell activation. NFAT is expressed in T cells and some tissues such as the nervous system, bone and epidermis, and recent data suggest that NFAT is involved in the skin’s responses to UV radiation. NFAT is predominantly located in an inactive hyperphosphorylated state in the cytoplasm, but when NFAT is dephosphorylated by phosphatase calcineurin, its import into the nucleus in order to regulate gene transcription [125]. The UV-A-induced oxidative stress leads to calcineurin activation, therefore, NFAT could be in an active form in response to low doses of UV-A radiation. While high doses of UV-A radiation induce phosphorylation of c-JUN through JNK, JNK bound to c-JUN may phosphorylate NFAT. These results suggest that NFAT in activate or inactivated state may be depending to different doses of UV-A [126]. Cyclooxygenase 2 (COX-2) protein levels become elevated in the epidermis of human skin following UV-B irradiation and also in squamous cell carcinomas and actinic keratosis. Heterozygous COX-2 knockout mice showed that COX-2 is required for the development of UV-induced skin tumors. The COX-2 promoter contains binding sites for NFAT, and in some reports, it has been shown that overexpression of COX-2 is dependent of NFAT, which has antiapoptotic effect in response to UV-B [127]. This result suggested that NFAT in an activated or inactivated state is depending of doses and UV wavelength radiation. UV radiation-induced NFAT may be negatively regulated by MAPK activation, while phosphatase activations in response to UV irradiation may regulate positively NFAT [128].

## 5. Deciphering Cellular Response to UV Radiation in Skin Using Genomic Tools

Sunburns are largely owing to UV-B radiation that is considered as the most successful inducer of damaging effects on epidermis, such as wrinkling, aging, keratosis, cancers of keratinocyte origin [129,130], as well as a potential contributing factor to the pathogenesis of melanoma. Several *in vit*ro models have been developed to study the molecular mechanism involved in human skin pigmentation and cancer [131,132], inflammation and DNA repair processes [133]. These models include mono-cultured cell lines, co-cultured melanocytes and keratinocytes, intact skin or suction blisters, as well as reconstructed epidermis.

DNA microarray technology has become one of the crucial tools for understanding the biological mechanisms associated with skin damage. One of the first studies showed that UV-B radiation affects the expression of at least 198 genes in normal epidermal keratinocytes cultures from human skin [44]. At 0.5–2 h after irradiation, alterations in gene expression of transcription factors, kinases, phosphatases, proteases, RNA processing enzymes, signal transducers, and cytoskeleton components, were notable, which suggests that keratinocytes have sensed DNA damage and changed their physiology to respond to UV stimulation. The early induced transcription factors included JUNB, JUND, c-FOS, ETR101, EGR1, HRY, and XBP-1. Their expression was relatively fast and short, which suggests that they could be activating the expression of DNA damage sensors and DNA repair factors. The overexpression of TAFII30, a TATA-box binding protein associated factor, could be related to the enhanced transcription observed for many genes. In contrast, UV irradiation induced a strong and persistent suppression of c-Myc, which is known to inhibit the expression of DNA damage-induced growth-arrest proteins. The regulation of kinases, phosphatases, small GTP binding proteins and their associated factors induced three RING3 proteins, which is consistent with the activation of intracellular signaling processes. It is known that the main signal-transducing mechanism directly induced by UV irradiation implies the participation of JNK and JNK-phosphorylated transcription factors [134–136]. The early suppression of the desmosomal proteins was noteworthy; since desmosomes keep cells closely attached one to another [137], their reduction may make easy the movement of keratinocytes, assembly of the cornified layer, and arrangement of the stratum corneum, which is the dead protective layer of skin. The most induced genes early after UV treatment include mitochondrial proteins (cytochrome c-1, cytochrome c oxidase subunit VIIb, and cytochrome b) and genes involved in energy generation (NADH dehydrogenase, mitochondrial ATP synthase, nucleoside-diphosphate kinase, an electron transfer flavoprotein, and α-enolase). In contrast, both transporters and gluconeogenic and lipogenic enzymes expression were repressed.

At 4–8 h, cells secreted growth factors, and produced cytokines and chemokines to alert the surrounding tissues to the UV damage. Specifically, five members of the IL-8 family were induced: IL-8, Gro-α, Gro-β, MDCNF and MIP2. These chemokines act as chemotactic and stimulate basophils, neutrophils, and macrophages, apparently promoting inflammatory response into UV-damaged tissue and activating melanocytes [138]. It has been demonstrated that keratinocytes do not produce interferon γ (IFNγ) but they have the capacity to respond to its presence inducing the expression of K17, the keratin in contractile epithelia [139]. In fact, at late time points after UV irradiation, IFNγ responsive genes were induced, such as p27, 17-kDa/15-kDa protein, IRF7A, hPA28β and IFNγ receptor accessory factor 1. At these times, genes for components of the cornified layer of skin were induced too, including differentiation markers, such as calgranulin, elafin, involucrin and S100 calcium-binding protein A13, and four small proline-rich proteins [140], which suggests that the epidermal responses to UV comprises the enrichment of stratum corneum through augmentation of the cornified, dead, protective layer of skin. It seems that one of the epidermal responses to UV is enrichment of stratum corneum.

A comparison of these data with other reports obtained from several time-course cDNA microarrays, revealed a very similar UV-B-regulated gene expression profile in keratinocytes [141–144]. Additional outcomes in epidermal keratinocytes showed the induction of neuroendocrine-immune-related genes involved in water and salt balance, prostaglandin synthesis, keratinocyte differentiation, as well as genes coding for stress effectors, cytokines and metalloproteinases [145]. Remarkably, HaCaT cells were able to respond to UV-B radiation in a similar way than healthy epidermal keratinocytes [146]. Among 840 genes tested, 192 changed their expression levels at different time points. The genes were clustered into four groups in self-organizing maps and classified into nine functional categories according to the affected biological processes. Those that were first up regulated and then returned to normal levels included genes related to the inhibition of cell growth and the proteasome processing. On the other hand, the expression of numerous genes involved in the cytoskeleton dynamic, signaling, metabolism and transcription were first down regulated or unchanged and then up regulated, reflecting the restoration of UV-B-damaged cellular activities. Another study using cDNA microarray technology elucidated the effect of a cytotoxic dose of UV-B on cancer-related genes in human epidermal keratinocytes (neonatal) at 1, 6, and 24 h post-radiation. Once verified that viability of the irradiated cells was 75% at 24 h post-irradiation, analysis of the hybridization signals revealed that 27 genes were regulated within 1 h after UV-B treatment. Moreover, 58 and 28 genes were regulated at 6 and 24 h post-irradiation, respectively. Various cytokeratins and transcription factors were up regulated within 1 h post-irradiation. After 6 h, expression of a variety of genes related to growth regulation (p21WAF1, notch 4, and smoothened), apoptosis (caspase 10, hTRIP, and CRAF1), DNA repair (ERCC1, XRCC1), cytokines (IL-6, IL-13, TGFβ, and endothelin 2), and cell adhesion (RhoE, and RhoGDI) were altered in human keratinocytes [147].

Interestingly, in a squamous cell carcinoma line, the effect of UV-B radiation contrasted from the response of normal human keratinocytes. Normal cells were found to be more resistant than carcinoma to UV-B-induced apoptosis, and this resistance was principally associated to the secretion of survival factors. The effect of UV-B on normal cells and carcinoma line induced the up regulation of 251 and 127 genes, respectively, and the down regulation of 322 and 117 genes, respectively, in these cultures. Although the response of keratinocytes to UV-B involved control of crucial checkpoint genes, such as p53, MDM2, p21(Cip1), DeltaNp63, as well as antiapoptotic and DNA repair genes, little or no change in the expression of these genes was detected in squamous cell carcinoma line [148]. Despite the fact that keratinocytes are the most plentiful skin cell type, they do not completely represent human skin *in vivo* because epidermis contains Langerhans cells, which are the resident antigen-presenting dendritic cells, Merkel cells, the mechanoreceptive cells related to sensory neurons, and melanocytes that provide pigments as defense from sunlight and comprise about 10% of all epidermal cells. Facultative pigmentation, as a consequence of physiological reaction to UV radiation and skin constitutive color, commits the epidermal pigmentation controlled by melanocytes and melanosome metabolism [149]. In addition, low doses of UV-B radiation have been shown to cause a greater increase in melanin synthesis in human co-culture models based on melanocyte and keratinocyte cells [150,151], than in mono-cultured melanocytes [152]. Even though the epidermis might thicken with prolonged UV exposure, the melanocytes undertake crucial physiological modifications, including bigger dendrite formation, melanogenesis, and melanosome translocation that are responsible for maintaining an adequate protection to minimize UV chronic damage [153].

Molecular approaches on UV responses have also advanced rapidly using primary melanocyte cultures and cultivated melanoma cells. It has been reported that UV exposure induces a G1 arrest in melanocytes which is in part due to p53 [154] and p16INK4a [155]. Notably, a polymorphism in p53 protein (Arg72Pro) is responsible for risk of melanoma [156]. In melanocytes, many molecules are induced by UV treatment [157–161]. The activated network contains transcription factors (Id1, N-oct3, p73, p53, Mitf), signaling kinases (JNK, ATR, p38, AKT), effectors for cell cycle control (p16INK4a, Gadd45, p21CIP), DNA reparation (Gadd45, Ddb2) and survival (Bax, Bcl-2) [162–164].

A relevant widespread study evaluated UV-B-mediated alterations in over 47,000 transcripts by microarrays assays, using primary melanocytes cultures as model. A set of p53 targets, including the cell cycle regulator CDKN1A (p21CIP), WNT pathway regulator DKK1 (dickkopf homolog 1), receptor tyrosine kinase EPHA2, growth factor GDF15, ferrodoxin-reductase (FDXR), p53-inducible protein (TP53I3), transcription factor ATF3, DNA repair enzyme DDB2, and β-adrenergic receptor ADBR2, were identified. Besides, a subgroup of neurite/axonal developmental genes was modulated, suggesting that melanocytic and neuronal arborization may have related mechanisms. Compared to melanomas, the basal level of many of these p53-responsive genes was greatly deregulated. Genes like CDKN1A, DDB2 and ADRB2, showed a decreased expression in melanomas, increasing the probability to perform a concomitant function in carcinogenesis events [165].

The role of UV-B radiation in skin cancer has been considerably emphasized. In contrast, the adverse effects of UV-A have often been dismissed and comparatively less studied. At present, many published articles showed the protagonist action of UV-A on skin carcinogenesis. Unlike UV-B, which is partially absorbed by the different epidermal layers and does not penetrate into the skin, UV-A rays affect the dermal compartment by radically decreasing the amount of superficial fibroblasts in epidermis [166]. The prevalence of UV-A mutations in the basal cell layer supports the essential role that UV-A may perform in the malignant alterations of human skin [167]. Besides, UV-A radiation induces epidermal lipid peroxidation, which stimulates Langerhans cells migration from the epidermis, thus contributing to UV-induced immunosuppression [168], as well as alteration of the expression of TNFα in keratinocytes [169].

Gene expression profiles in melanocytes and other types of skin cells submitted to different types of UV have been recently reported and potential previously unidentified factors involved in UV-induced responses of human skin have been identified. Whole human genome microarray analyses were used to characterize human skin *in situ* and examine how melanocyte specific proteins and paracrine melanogenic factors are regulated by repetitive exposure to different types of UV. The results showed that UV-B radiation elicited a dramatic increase in a large number of genes involved in pigmentation, as well as in other cellular functions, while UV-A had little or no effect on these processes [170]. Another study using microarray technology confirmed that UV-A regulates genes involved in extracellular matrix homeostasis, oxidative stress, heat shock responses, cell growth, inflammation and epidermal differentiation [171]. In addition, modulations for genes previously unknown to be involved in the UV-A response, such as PLAB, EPO, HBEGF, IGF1 and TGF-α, all corresponding to growth factors family were described. Furthermore, this work also revealed that broad-spectrum sunscreen application prior to UV-A exposure abrogated or significantly reduced these effects, because it was able to normalize gene expression in both fibroblasts and keratinocytes [171].

One last interesting issue is that most experimentation characterizing the molecular effects of sun exposure in skin has generally involved solar simulators. In most cases, the aim was to explore a very drastic UV radiation panorama, which is barely found in routinely open-air activities. A non-extreme UV spectrum denoted as “daily UV radiation” (D-UVR) with a higher UV-A than UV-B radiation ratio, has consequently been considered in several experimental projects. The biological impact of exposure to low physiological doses of D-UVR on a three-dimensional reconstructed skin model has been studied. In such conditions, skin morphological alterations could only be identified afterward the highest dose of D-UVR. Outcomes were focused on oxidative stress induced by D-UVR. A cell type differential response was observed: it was more rapid in fibroblasts, with a bulk of inductions and high levels of modulation, in contrast to keratinocyte behavior. Results evidenced a higher sensitivity in response to oxidative stress in dermal fibroblasts located at the bottom of epidermal layers, giving new insights into the skin molecular processes happening as a consequence of daily UV exposure [172].

In conclusion, DNA microarrays assays have emerged as the golden technique in the overall analysis of gene expression, and their achievements have bettered even the most positive expectations. Their scopes include the understanding of molecular events associated with unhealthy epidermis and skin carcinogenesis. Fortunately, these findings shall lead us to the creation and improvement of medical therapies and resources to develop the function of healthy skin, as well as the design of new politics of prevention.

## 6. Concluding Remarks

As described above, the cellular response to UV radiation involves multiple and specific signal transduction pathways and transcription factors. Deciphering the complex interplay between signaling kinases and transcription factors effectors in the control of gene expression after UV radiation is imperative to develop novel therapeutic strategies to overcome skin damage. Progress in understanding the multitude of mechanisms induced by UV-exposure could lead to the identification and potential development of specific inhibitors for the prevention and control of skin photoaging and carcinogenesis.

## Figures and Tables

**Table 1:** Protein kinases and transcription factors activated by specific UV wavelengths.

	MAPK pathway	PI-3K pathway	Transcription factors
UV-A	EGFR, SRC, RAS, RAF, ERK1/2, p70^s6k^ and p90^RSK^	ATM	c-JUN, Nrf2, NFAT
UV-B	PKC, JNK, p38 kinase, RSK2, MSK1	EGFR, PI-3K, Akt, p70^s6k^ and p90^RSK^	c-FOS, NF-κB NFAT
UV-C	EGFR, SRC, ERK, p38 kinase JNK1/2	ATR	AP-1

## Abbreviations

UV: Ultraviolet
ROS: Reactive Oxygen Species
TNFR: Tumor Necrosis Factor Receptor
MAPK: Mitogen-Activated Protein Kinases
PI-3K: Phosphoinositide 3-Kinases
EGFR: Epidermal Growth Factor Receptor
PKC: Protein Kinase C
ATM: Ataxia Telangiectasia Mutated
ATR: ATM-Related
JNK: c-Jun NH_2_-Terminal Kinases
RSK2: p90 Ribosomal S6 Kinase 2
MSK1: Mitogen-and Stress-Activated Protein Kinase
MKPs: Mitogen-Activated Protein Kinase Phosphatases
PP2A: Phosphatase 2A
Wip1: Wild-Type p53-Induced Phosphatase
PTP: Protein-Tyrosine Phosphatase
PIP2: Phosphatidylinositol-(4,5)-Bisphosphate
AP-1: Activator Protein-1
TLRs: Toll-Like Receptors
NF-κB: Nuclear Factor kappa B
NIK: NF-κB Inducing Kinase
IKK: IκB Kinase
NEMO: NF-κB Essential Modulator
CK2: Casein Kinase II
LTR: Long Terminal Repeat
HIV-1: Human Immunodeficiency Virus-1
eIF2α: Eukaryotic Translation Initiation Factor 2α
PERK: Protein Kinase Like Endoplasmic Reticulum Kinase
EGCG: (−)-Epigallocatechin 3-gallate
bFGF: Basic Fibroblast Growth Factor
MMP-1: Matrix Metalloproteinase-1
ODNs: Oligodeoxynucleotides
TPA: Phorbol Ester 12-*O*-Tetradecanoylphorbol-13-Acetate
MAF: Musculo-Aponeurotic Fibrosarcoma
Nrf2: NF-E2-Related Factor 2
ARE: Antioxidant Responsive Element
KGF: Keratinocyte growth factor
NFAT: Factor of Activated T Cells
COX-2: Cyclooxygenase 2
